# Application of shear stress for enhanced osteogenic differentiation of mouse induced pluripotent stem cells

**DOI:** 10.1038/s41598-022-21479-8

**Published:** 2022-11-08

**Authors:** Phoonsuk Limraksasin, Praphawi Nattasit, Jeeranan Manokawinchoke, Watcharaphol Tiskratok, Naruephorn Vinaikosol, Hiroko Okawa, Chalida Nakalekha Limjeerajarus, Nuttapol Limjeerajarus, Prasit Pavasant, Thanaphum Osathanon, Hiroshi Egusa

**Affiliations:** 1 Division of Molecular and Regenerative Prosthodontics, Tohoku University Graduate School of Dentistry, 4-1 Seiryo-machi, Aoba-ku, Sendai, Miyagi 980-8575 Japan; 2 Dental Stem Cell Biology Research Unit, Faculty of Dentistry, Chulalongkorn University, Bangkok, 10330 Thailand; 3 Center of Excellence for Regenerative Dentistry and Department of Physiology, Faculty of Dentistry, Chulalongkorn University, Bangkok, 10330 Thailand; 4 Center of Excellence for Regenerative Dentistry and Department of Anatomy, Faculty of Dentistry, Chulalongkorn University, 34 Henri-Dunant Rd. Pathumwan, Bangkok, 10330 Thailand; 5 Office of Research Affairs, Faculty of Dentistry, Chulalongkorn University, Bangkok, Thailand; 6 Research Center for Advanced Energy Technology, Faculty of Engineering, Thai-Nichi Institute of Technology, Bangkok, Thailand; 7 Center for Advanced Stem Cell and Regenerative Research, Tohoku University Graduate School of Dentistry, 4-1 Seiryo-machi, Aoba-ku, Sendai-city, 980-8575 Japan

**Keywords:** Biotechnology, Cell biology, Developmental biology, Stem cells

## Abstract

The self-organizing potential of induced pluripotent stem cells (iPSCs) represents a promising tool for bone tissue engineering. Shear stress promotes the osteogenic differentiation of mesenchymal stem cells, leading us to hypothesize that specific shear stress could enhance the osteogenic differentiation of iPSCs. For osteogenesis, embryoid bodies were formed for two days and then maintained in medium supplemented with retinoic acid for three days, followed by adherent culture in osteogenic induction medium for one day. The cells were then subjected to shear loading (0.15, 0.5, or 1.5 Pa) for two days. Among different magnitudes tested, 0.5 Pa induced the highest levels of osteogenic gene expression and greatest mineral deposition, corresponding to upregulated connexin 43 (Cx43) and phosphorylated Erk1/2 expression. Erk1/2 inhibition during shear loading resulted in decreased osteogenic gene expression and the suppression of mineral deposition. These results suggest that shear stress (0.5 Pa) enhances the osteogenic differentiation of iPSCs, partly through Cx43 and Erk1/2 signaling. Our findings shed light on the application of shear-stress technology to improve iPSC-based tissue-engineered bone for regenerative bone therapy.

## Introduction

Owing to its osteoinductive properties, stem cell-based therapy for bone regeneration is expected to improve bone healing in the clinic^1^. Clinical applications of stem cell therapy, especially in bone augmentation, generally involve autologous bone grafts or allogenic bone graft materials containing mesenchymal stem cell (MSC)-enriched growth factors. However, these approaches may have certain disadvantages when applied to critical-size defects such as donor site morbidity, fibrous tissue encapsulation, material allergy, as well as MSC migration and death^2^. In contrast, induced pluripotent stem cells (iPSCs) have unlimited proliferation capacity and could be a stem cell reservoir for in vitro tissue engineering. In addition, iPSCs possess pluripotency and self-organizing potential, can differentiate into three germ layers (endoderm, mesoderm, ectoderm), and can form 3-dimensional (3D) tissue or organs without the use of a scaffold. Hence, iPSCs could be a promising source for bone tissue engineering. The development of methods for improving iPSC osteogenic differentiation represents an attractive research line within the field of regenerative medicine. Our group previously established osteogenic induction differentiation in both adherent^3^ and 3D-culture^4^ of mouse iPSCs. However, undifferentiated iPSCs still remained in the culture. Therefore, the osteogenic differentiation of iPSCs requires greater stimuli to guide sufficient iPSC differentiation into osteogenic cells.

Mechanical stimuli, particularly those derived from shear, compressive, and tensile forces, play roles during embryogenesis/organogenesis. There have extensive studies on compressive force enhancing the osteogenic differentiation of human periodontal ligament stem cells (PDLCs) and the underlying mechanisms^5^. In contrast, cells in developing bone, cartilage, cardiac, and blood vessels are regularly exposed to physiological fluid shear stress^6^. Previous studies have shown that shear stress enhances the osteogenic differentiation of mesenchymal stromal cells^7^, osteoblasts^8^, and osteocytes^9^ in different manners depending on the magnitude, duration, as well as frequency of the applied shear force. In addition, our previous study showed that shaking culture could facilitate the generation of osteogenic^4^, osteochondrogenic^10^, and chondrogenic^11^ constructs. However, the mechanisms underlying this phenomenon are poorly understood. It should be noted that shaking culture using a see-saw shaker or bioreactor does not include only shear stress to those constructs, but also other types of forces such as compressive and impact forces.

Recent trends in organoid technology, as well as bone tissue engineering for regenerative medicine, rely on iPSC self-organization, which still requires a strong factor for osteogenic differentiation. A previous study showed that laminar shear force could regulate lineage specification in mouse embryonic stem cells (ESCs)^6^, promoting a hematopoietic stem cell^12^ and an endothelial cell-like phenotype^13^ during early ESC differentiation. Shear force has been widely studied for the osteogenic differentiation of stem/bone cells. Although shear stress is attractive for improving bone tissue engineering, its effects on the osteogenic differentiation of iPSCs remain unclear. Therefore, we hypothesized that specific shear stress could enhance mouse iPSC osteogenic differentiation. To test this hypothesis, shear stress was applied to the adherent embryoid body (EB) culture under osteogenic induction using a shear stress loading apparatus, subjecting cells to continuous shear force application^14^.

## Results

### Effects of continuous shear stress on mouse iPSC viability, proliferation, and pluripotency

**Figure 1:**
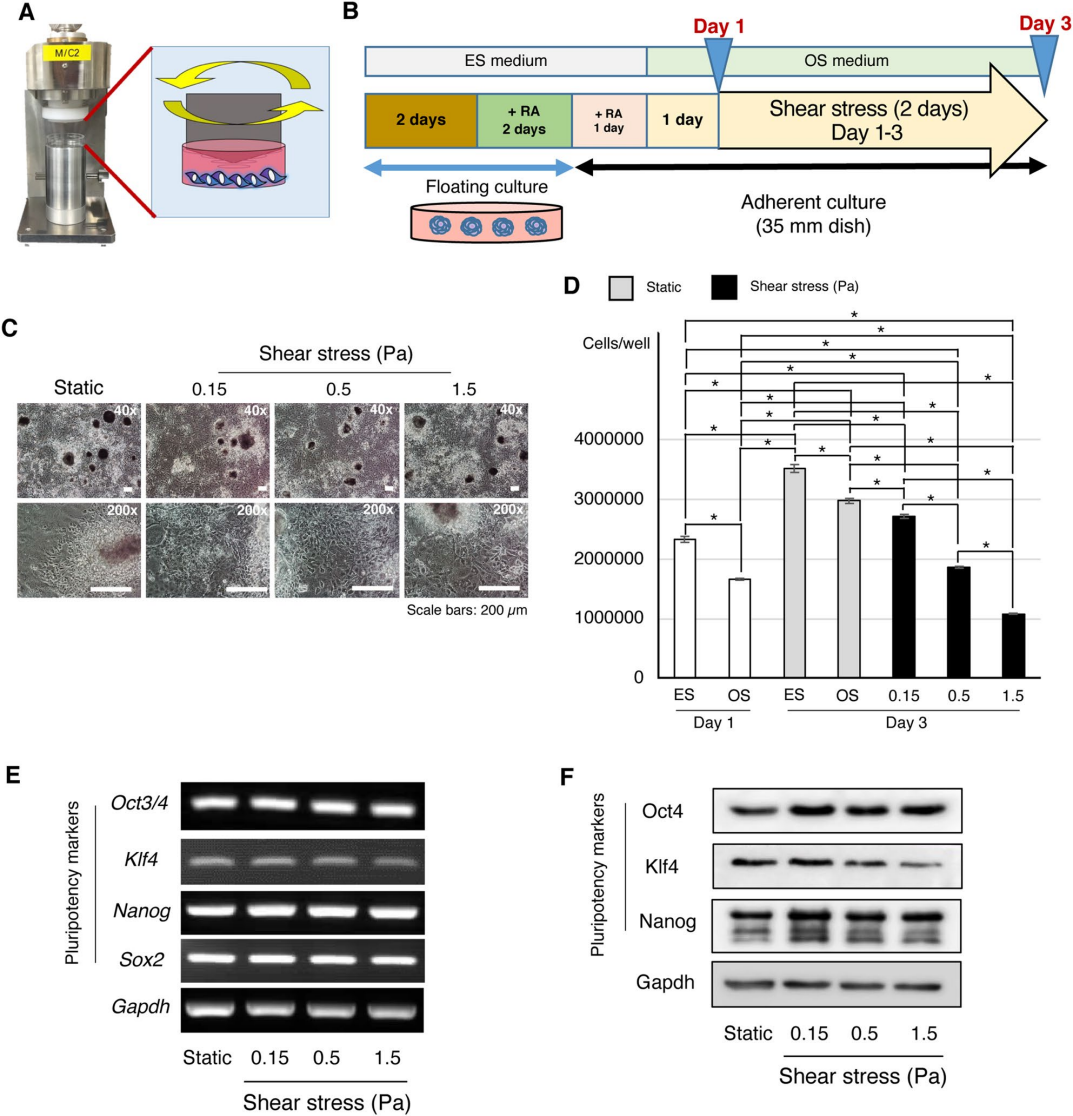
Continuous shear stress influenced the proliferation and pluripotency of iPSCs. (**A**) A novel shear stress loading apparatus was used in this study. (**B**) Osteogenic induction method. In brief, the embryoid bodies (EBs) were formed for two days and then maintained in iPSC growth (ES) medium supplemented with retinoic acid (RA), followed by adherent culture in osteogenic induction (OS) medium for one day prior to the application of shear force (0.15, 0.5, and 1.5 Pa) for 48 h. (**C**) Cell morphology and viability of static culture and shear loading (0.15, 0.5, and 1.5 Pa) on Day 3 of osteogenic induction were observed under a phrase-contrast microscope. The magnifications of upper and lower panels are 40× and 200×, respectively. Scale bars: 200 μm (**D**) WST-1 assay was performed to detect cell viability after shear force application (0.15, 0.5, and 1.5 Pa) for 48 h (Day 3). 3-day static culture in ES medium or OS medium was used as a control for cell viability. The cells in OS medium before applying shear stress (OS; Day 1) was used as a control to detect cell proliferation after applying shear stress for 48 h (Day 3). *: *P* < 0.05, ANOVA with Tukey’s multiple comparison test. Data represent the mean ± SD (n = 3). (**E**) Reverse transcription-polymerase chain reaction (RT-PCR) was performed to detect mRNA expression levels of pluripotency genes *Oct3/4*, *Klf4*, *Nanog*, and *Sox2*. The expression of *Gapdh* was used as an internal control. Original gels are presented in Supplementary Fig. 4A. (**F**) Western blot analysis was performed to evaluate pluripotency protein markers Oct4, Klf4, and Nanog. Gapdh was used as a loading control. Original blots are presented in Supplementary Fig. 4B.

Mouse gingiva-derived iPSCs^15^ were subjected to osteogenic induction as per a method previously reported by our group^3^. After maintenance in osteogenic induction medium for one day, three different magnitudes of shear stress (0.15, 0.5, and 1.5 Pa)^6^ were applied to adherent iPSCs using a custom-designed shear stress loading system (Fig. 1A)^17^ for another 2 days (Fig. 1B). The magnitudes tested did not obviously affect cell viability nor the heterogeneous characteristics of cell outgrowth from adherent EBs (Fig. 1C). After maintenance in osteogenic induction medium for one day (Day 1 of induction), cell proliferation was significantly reduced compared to that in growth medium (ES medium) (Fig. 1D). After maintaining the cells under static condition for another two days (Day 3), the cell number in osteogenic induction medium was significantly lower than that in ES medium. On Day 3, the cell number under shear stress application (0.15, 0.5, and 1.5 Pa) was significantly lower than that in the static group under osteogenic induction medium culture, decreasing in a force-dependent manner. The gene expression of Octamer-binding transcription factor 4 (*Oct3/4*), Sex-determining region Y-box 2 (*Sox2*) and *Nanog* slightly increased after shear stress application for two days, whereas Krüppel-like factor 4 (*Klf4*) expression slightly decreased in a force-dependent manner (Fig. 1E and Supplementary Fig. 1A). The protein expression of Nanog and Oct4 increased in the shear stress loading groups (Fig. 1F and Supplementary Fig. 1B). Klf4 protein expression decreased in the 0.5 and 1.5 Pa shear force loading groups, while a slight increase of Klf4 expression was observed under shear stress loading at 0.15 Pa (Fig. 1F and Supplementary Fig. 1B).

### Effects of continuous shear stress on the osteogenic differentiation of mouse iPSCs

**Figure 2:**
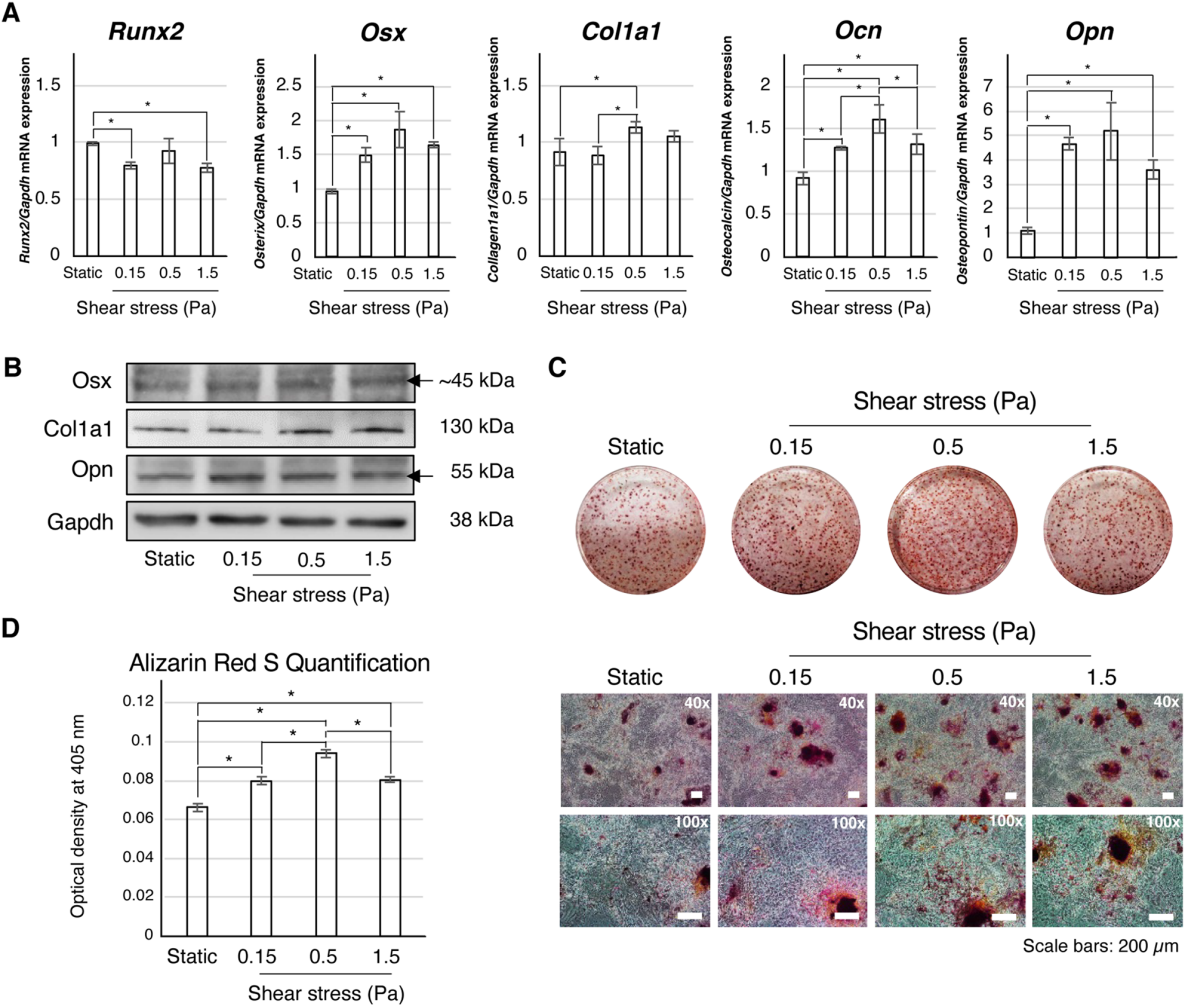
Continuous shear stress enhanced the osteogenic differentiation of iPSCs in a force-independent manner. iPSCs were cultured in OS medium with 0.15, 0.5, and 1.5 Pa of shear force application for 48 h prior to (**A**) real-time RT-PCR, (**B**) western blotting, and (**C**) Alizarin Red S staining analyses. Static culture of iPSCs in Os medium was collected for evaluation at the same time point as a control. (**A**) Real-time RT-PCR was performed to detect the expression of osteogenic genes *Runx2*, *Osx*, *Col1a1*, *Ocn*, and *Opn*. *Gapdh* was used as an internal control. *: *P* < 0.01, ANOVA with Tukey’s multiple comparison test. Data represent the mean ± SD (n = 3). (**B**) Western Blotting analysis of osteogenic markers Osx, Col1a1, and Opn. Gapdh was used as a loading control. Original blots are presented in Supplementary Fig. 5. (**C**) Positive Alizarin Red S staining is shown in red. The magnifications of upper and lower panels are 40 × and 100x, respectively. Scale bars: 200 μm. (**D**) Alizarin Red S staining was quantified based on absorbance at 405 nm. *: *P* < 0.05, ANOVA with Tukey’s multiple comparison test. Data represent the mean ± SD (n = 3).

We explored the effect of different shear force magnitudes on osteogenic expression. Real-time PCR analysis showed that osterix (*Osx*), osteocalcin (*Ocn*), and osteopontin (*Opn*) were significantly upregulated under shear stress. *Ocn* expression was highest in the 0.5 Pa group (Fig. 2A). Collagen 1a1 (*Col1a1*) expression was significantly increased only in the 0.5 Pa group relative to the static group. In contrast, the expression level of *Runx2* was suppressed in the 0.15 and 1.5 Pa groups, but not in the 0.5 Pa group, when compared to the static group (Fig. 2A). The expression of osteogenic-related proteins Osx, Col1a1, and Opn were higher in the shear stress application group than in the static group (Fig. 2B). Positive Alizarin Red S (ARS) staining was observed in the EB region of all groups (Fig. 2C). In contrast, positive staining of cell outgrowth areas was observed in the shear stress loading group (Fig. 2C). The quantification of ARS staining revealed a significant increase in mineralization relative to the static group, with highest mineralization observed in the 0.5 Pa group (Fig. 2D). Furthermore, the pretreatment of osteogenically-induced iPSCs with shear stress for 2 days before the transplantation enhanced in *vivo bone* formation in critical-size calvaria defects (Supplementary Fig. 2).

### Effects of shear stress duration on osteogenic differentiation of mouse iPSCs

**Figure 3:**
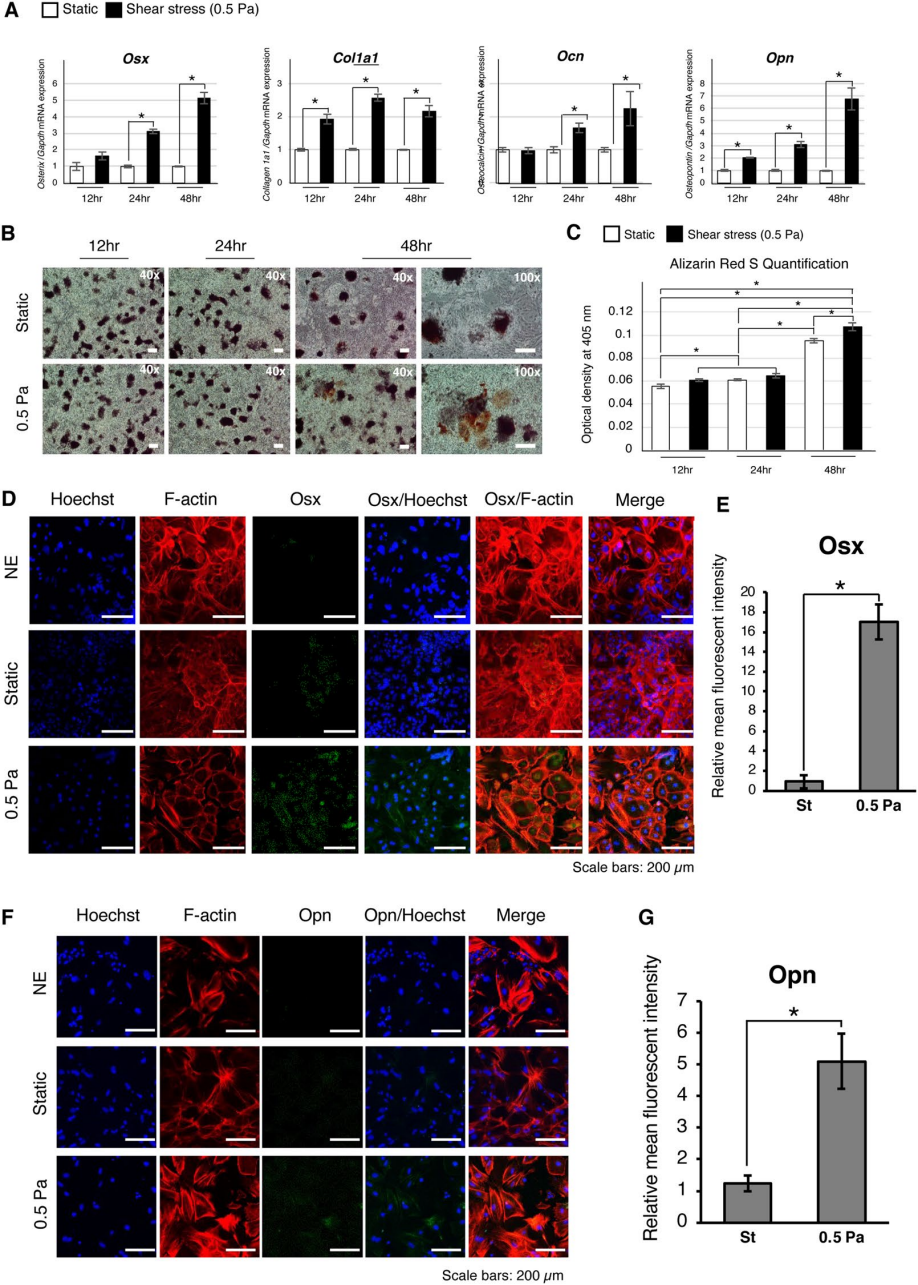
Continuous shear stress enhanced the osteogenic differentiation of iPSCs in a time-dependent manner. Evaluation of osteogenic differentiation under 0.5 Pa; iPSCs were maintained in OS medium for one day prior to the application of 0.5 Pa of shear stress for 12, 24, and 48 h, respectively. The control static culture in OS medium was collected at the same time point for evaluation (**A**) Relative gene expression of osteogenic markers (*Osx*, *Col1a1*, *Ocn*, and *Opn*) was performed by Real-time RT-PCR. *: *P* < 0.05, Student’s *t*-test. Representative data from three independent experiments are shown (mean values ± SD: n = 3) (**B**) Alizarin Red S staining (red) was performed. The magnifications of upper and lower panels are 40 × and 100x, respectively. Scale bars: 200 μm. (**C**) Quantification of Alizarin Red S staining to evaluate mineralization level. *: *P* < 0.05, ANOVA with Tukey’s multiple comparison test. Representative data from three independent experiments are shown (mean values ± SD: n = 3). Immunofluorescence staining was performed to detect the nuclear localization of (**D**) Osx (green) and the expression of (**F**) Opn (green). Normal mouse IgG was used as negative control (NC). The cytoskeleton (F-actin; red) and nuclei (blue) were stained with rhodamine-phalloidin and Hoechst, respectively. Scale bars: 200 μm. The relative mean fluorescence intensity of (**E**) Osx and (**G**) Opn was quantified by ImageJ software. *: *P* < 0.05, Student’s *t*-test. Data represent the mean ± SD (n = 3).

*Osx* and *Ocn* were upregulated after 24 h of shear stress application (Fig. 3A). The fold change in *Osx* expression relative to the static group further increased after 48 h of exposure. *Col1a1* and *Opn* were significantly upregulated after 12 h of shear stress, with the latter further increasing to 3- and 7-fold at 24 h and 48 h, respectively, while the fold change of *Col1a1* expression remained at 2–2.5 for all time points (Fig. 3A). No positive staining was noted at 12 nor 24 h in the static group. In contrast, positive ARS staining was observed in the outgrowth cell area of the 0.5 Pa group (Fig. 3B). ARS quantification revealed that mineral deposition in the 0.5 Pa group significantly higher than that in the static group for 48 h (Fig. 3C). The expression and nuclear localization of Osx was enhanced after applying shear stress (0.5 Pa) for 48 h (Fig. 3D and E). Expression of Opn protein was higher in the 0.5 Pa group relative to the static group (Fig. 3F and G).

### Involvement of connexin 43 (Cx43) and Trpm7 in the shear stress-induced osteogenic differentiation of mouse iPSCs

**Figure 4:**
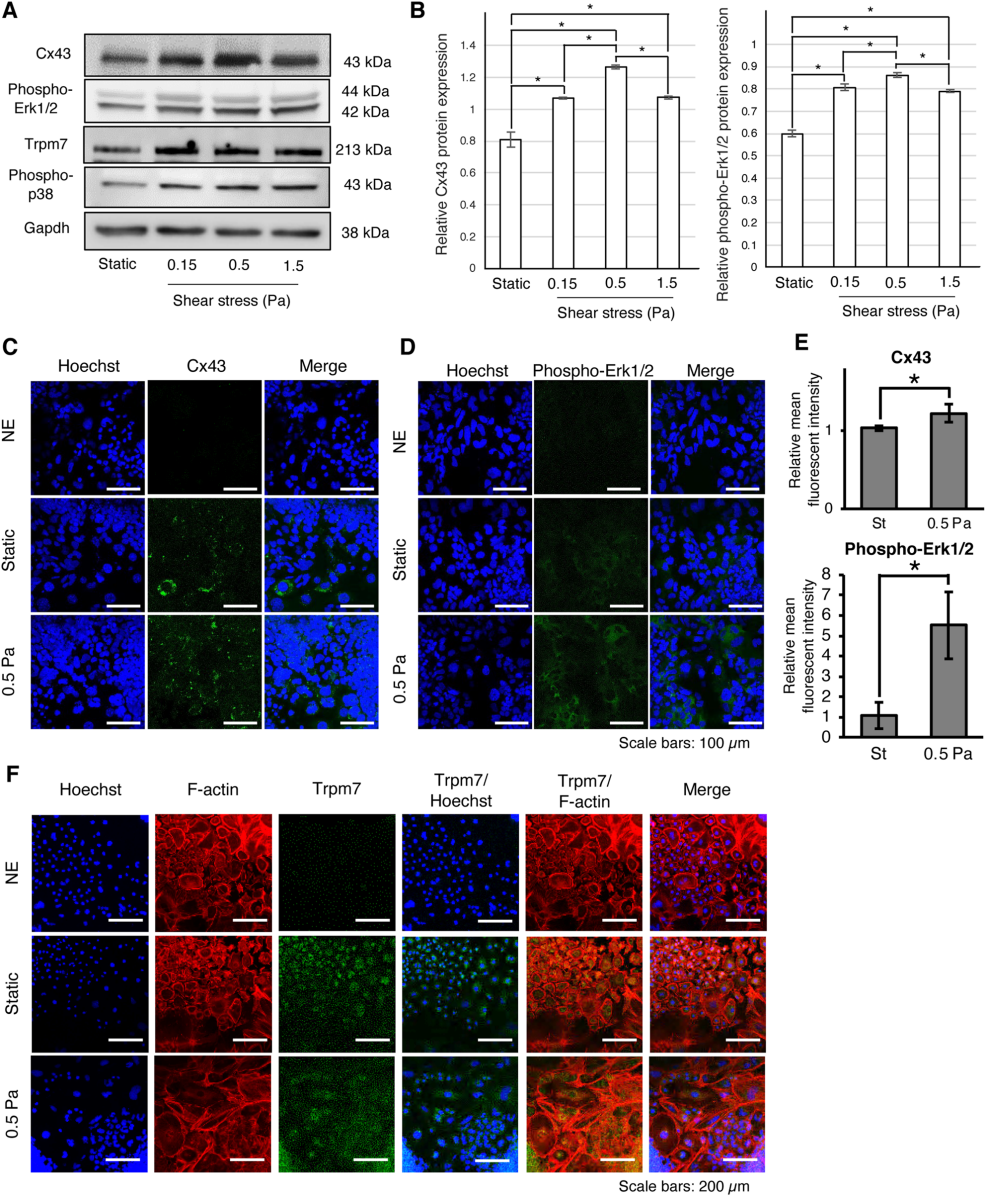
Mechanisms involved in continuous shear stress-enhanced osteogenic differentiation of iPSCs. iPSCs were analyzed after being subjected to shear stress for 48 h. (**A**) Western blot analysis of cell surface channels Connexin 43 (Cx43) and Trpm7 as well as the phosphorylation of downstream factors including Erk1/2 (phospho-Erk1/2) and p38 (phospho-p38). Original blots are presented in Supplementary Fig. 6. (**B**) Quantitative analysis of Cx43 and phospho-Erk1/2 expression using ImageJ software. The expression levels were normalized to Gapdh expression. *: *P* < 0.05, ANOVA with Tukey’s multiple comparison test. Representative data from three independent experiments are shown (mean values ± SD: n = 3). Immunofluorescence analysis was performed to detect the protein expression and localization of (**C**) Cx43 (green), (**D**) phospho-Erk1/2 (green), and (**F**) Trpm7 (green). Normal mouse IgG and normal rabbit IgG were used as a negative control (NC). The cytoskeleton (F-actin; red) and nuclei (blue) were stained using rhodamine-phalloidin and Hoechst, respectively. Scale bars: (**C** and **D**) 100 μm, (**F**) 200 μm. (**E**) The relative mean fluorescence intensity of Cx43 and phospho-Erk1/2 was quantified by ImageJ software. *: *P* < 0.05, Student’s *t*-test. Data represent the mean ± SD (n = 4).

A previous report demonstrated that shear stress enhanced the osteogenic differentiation of mouse MSCs^16^ and human PDLCs^17^ through the regulation of cell surface channels Trpm7 and Cx43 via the p38 and Erk1/2 pathways, respectively. After applying shear stress for two days, the expression of Tprm7, Cx43, phospho-Erk1/2, and phospho-p38 proteins enhanced in the 0.15, 0.5, and 1.5 Pa shear stress application groups compared to the static group (Fig. 4A). In addition, the highest Cx43 expression was observed in the 0.5 Pa group (Fig. 4A). Quantitative analysis of the band density for the Western blotting analysis showed significantly higher Cx43 and phosphorylated Erk1/2 expression in the 0.5 Pa group (Fig. 4B). Cx43 expression was observed surrounding nuclei in the static group, whereas greater Cx43 expression in the 0.5 Pa group was observed surrounding the cell membrane (Fig. 4C and E). Phosphorylation of Erk1/2 in the cytoplasm of cells was enhanced by the shear stress (0.5 Pa) (Fig. 4D and E). Trpm7 expression in the static group was detected mainly surrounding nuclei, while Trpm7 channel expression in the 0.5 Pa group was observed throughout cells (Fig. 4F).

### Involvement of the Erk1/2 signaling pathway in the shear stress-induced osteogenic differentiation of mouse iPSCs

**Figure 5:**
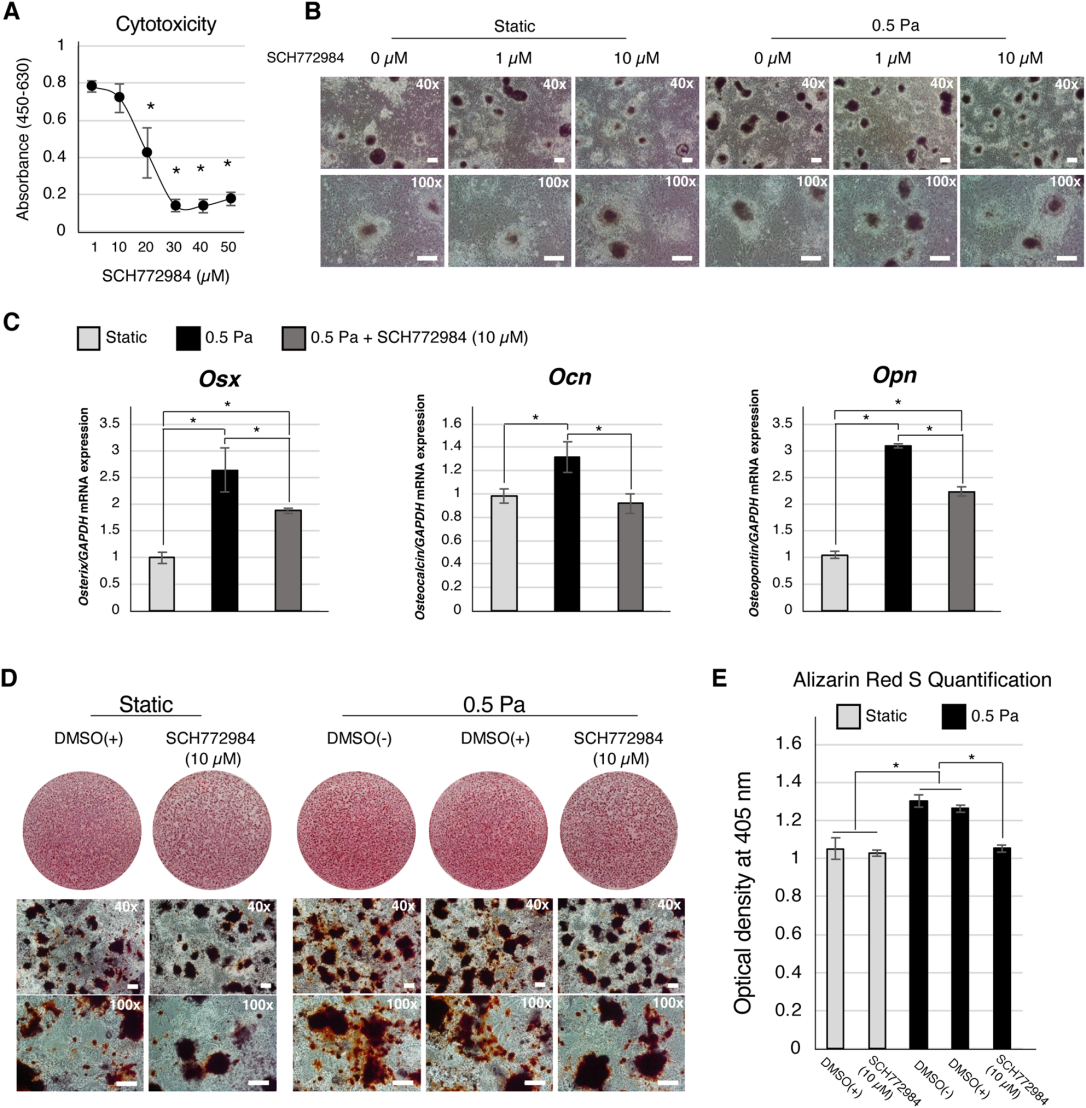
Inhibition of Erk1/2 activity suppressed the shear-stress enhanced-osteogenic differentiation of iPSCs. (**A**) WST-1 assay was performed to evaluate the cytotoxic effects of Erk1/2 phosphorylation inhibitor SCH772984 at various concentrations. *: *P* < 0.05, ANOVA with Dunnett’s correction for multiple comparisons. Representative data from three independent experiments are shown (mean values ± SD: n = 3). **(B**) Morphology and viability of osteogenically-induced iPSCs were observed under a phrase-contrast microscope. The magnifications of upper and lower panels are 40× and 100×, respectively. Scale bars: 200 μm. (**C**) Real-time RT-PCR analysis was performed to evaluate the expression of osteogenic genes (*Osx*, *Col1a1*, *Ocn*, and *Opn*). *: *P* < 0.05, ANOVA with Tukey’s multiple comparison test. Representative data from three independent experiments are shown (mean values ± SD: n = 3). (**D**) Alizarin Red S staining was performed to evaluate mineral deposits. After treatment with inhibitor during the application of 0.5 Pa shear force application, the cells were maintained in osteogenic induction medium for another five days prior to Alizarin Red S staining. The magnifications of upper and lower panels are 40× and 100×, respectively. Scale bars: 200 μm. (**E**) Quantitative analysis of Alizarin Red S staining. *: *P* < 0.05, ANOVA with Tukey’s multiple comparison test. Representative data from three independent experiments are shown (mean values ± SD: n = 3).

Cytotoxicity assays revealed no significant difference between treatment with 1 μM and 10 μM SCH772984, an Erk1/2 phosphorylation inhibitor. Higher concentrations (20, 30, 40, and 50 μM) resulted in significantly lower cell viability (Fig. 5A). Heterogeneous cell morphology was observed in the static and 0.5 Pa groups as well as under treatment with 1 μM and 10 μM SCH772984 (Fig. 5B). In the static culture group, treatment with 1 μM SCH772984 for 24 h did not affect osteogenic marker gene (*Osx* and *Ocn*) expression (Supplementary Fig. 1B). However, only *Osx* gene expression significantly decreased after treatment with 10 μM SCH772984 (Supplementary Fig. 1B). In contrast, after applying shear stress (0.5 Pa) for 24 h, *Osx*, *Ocn*, and *Opn* were upregulated. Further, the upregulation was significantly suppressed in the inhibitor-treated group (Fig. 5C). After applying shear stress for one day, the cells were maintained under static conditions for another 5 days prior to ARS staining. Enhanced mineral deposits were observed in the outgrowth cell area of the shear stress group (Fig. 5D). Treatment with 10 μM SCH772984 inhibited mineral deposition during shear stress application. Quantitative analysis of Alizarin Red S revealed significantly enhanced mineral deposition in the shear stress application group compared to the static group (Fig. 5E). No significant difference was observed between the inhibitor treatment group and the static group.

## Discussion

Mechanical stimulation has been reported to affect the growth and differentiation of stem cells. Our group previously demonstrated that intermittent compressive force promotes proliferation and suppresses apoptosis in RA-treated embryoid bodies of mouse iPSCs^18^. This implies a role for mechanical stimuli in promoting iPSC proliferation. However, our present study revealed that shear stress inhibited cell proliferation after two-day continuous application (Fig. 1D), which appears to contradict the previous report. It is possible that the culture conditions, including the medium, force type, and force application duration, differed from those in the previous report. The suppression of cell proliferation was observed in osteogenic induction medium compared to ES medium. This may imply greater cell differentiation in osteogenic induction medium, which in turn suppresses proliferation. After applying shear stress for two days, the suppression of cell proliferation occurred in a force-dependent manner. The greatest suppression of proliferation was observed in the 1.5 Pa group, while the highest number of cells was observed under 0.15 Pa. However, the cell number observed in the 1.5 Pa group was significantly lower than before applying force (Day 1), suggesting cell death or detachment during shear force application. A previous report showed that 1.5 Pa did not disturb the viability of mouse ES cells under laminar shear stress^6^, and the decrease in cell number may be attributed to the detachment of cells or EBs. In addition, our adherent EB culture, which is different from a previously reported single-cell monolayer culture with fibronectin coating^6^, might exhibit weaker adherence and, thus , easier detachment. Although 0.15 Pa and 0.5 Pa induced a decrease in cell number when compared to the static group on Day 3, the number of cells was still higher than that on Day 1, suggesting suppressed cell proliferation.

Stem cell multipotency has been reported to be restored by mechanical stimuli in 3D cultures. A previous study demonstrated that shaking culture restored the multipotency of mouse^19^ and human MSC spheroids after long-term expansion ex vivo^20^. In an iPSC study, mechanical stimulation using equiaxial stretching enhanced reprogramming efficiency, but did not enhance the transduction rate^21^. In addition, a transient increase in pluripotent marker gene expression was observed when iPSC constructs were subjected to shaking culture^10^. Although little evidence is available, mechanical stimuli seemed to influence the stemness, multipotency, and pluripotency of stem cells. Our results demonstrated that shear stress enhanced Nanog and Oct4 gene (Fig. 1E) and protein (Fig. 1F) expression. As our adherent EB culture contained heterogeneous cell types, the enhanced pluripotency under shear stress possibly occurred as a result of residual undifferentiated iPSCs. In contrast, Nanog, Oct4, and Sox2 are naturally expressed in adult stem cells, such as MSCs^22^. The Nanog and Oct4 expression under shear stress (Fig. 1F) might post the iPSC-derived MSCs. Klf4 is not only a pluripotent marker but is also expressed in mesenchymal cells of the skeletal primordia during early embryonic development, with its expression diminishing postnatally^23^. In addition, the expression and role of Klf4 have been reported in various tissues and organ systems, such as the intestine, eye, and skin. The attenuation of Klf4 expression in 0.5 and 1.5 Pa at this stage may imply osteogenic differentiation. In contrast, the slight increase of Klf4 expression in the 0.15 Pa group may be due to an increase in pluripotency or the differentiation of other Klf4-expressing cells. However, further studies, i.e., FACS analysis, are required to clarify this phenomenon whether these pluripotent genes’ expression is a result of residual undifferentiated iPSCs or MSCs.

Several studies have demonstrated that mechanical forces induce the differentiation and maturation of stem cells toward bone^4,24,25^ and cartilage cells^11,26–28^. Laminar shear stress mediates early lineage specification of single-cell monolayer-cultured mouse ES cells^6^. A previous report showed that a higher magnitude (1.5 Pa) enhanced ectodermal differentiation, whereas a lower magnitude (0.15 Pa) inhibited mesodermal differentiation. In addition, a short duration of force application (one day) enhanced ectodermal differentiation, whereas a longer duration (four days) promoted mesodermal differentiation. Our results showed that shear stress enhanced osteogenic differentiation of iPSCs. 0.5 Pa induced the greatest osteogenic cell differentiation (Fig. 2A and B) and mineralization levels (Fig. 2C and D). Osteogenic genes were significantly upregulated by shear stress, except for *Col1a1* expression, which was enhanced by 0.5 Pa of shear stress (Fig. 2A). The highest *Ocn* expression was observed in the 0.5 Pa group. Enhanced osteogenic differentiation of iPSCs was confirmed by an increase in osteogenic protein expression (Fig. 2B). Interestingly, the highest mineralization was observed under 0.5 Pa of applied force (Fig. 2C and D), wherein *Ocn* expression was also highest. Type I collagen and osteocalcin are major components of bone tissue^29,30^. The co-expression of these two factors in osteogenically-induced iPSCs suggested that 0.5 Pa might be the optimal magnitude for inducing the osteogenic differentiation of iPSCs. The application of 0.5 Pa of shear stress for one day had no effect on cell viability (data not shown). Although the expression level of Co1a1 gene and protein was not different between 0.5 and 1.5 Pa, the higher *Ocn* expression in 0.5 Pa could suggest more mature osteoblasts and thus demonstrated the higher mineralization in 0.5 Pa compared to 1.5 Pa group. The upregulation of Opn, in part, can indicate osteogenic differentiation; however, mechanical loading has been reported to directly control Opn expression by targeting a shear stress responsive element (SSRE; GAGACC) in the Opn promoter^31^. It is possible that the upregulation of Opn in 0.15 Pa group to the same level as 0.5 Pa group may be through this mechanism; however, further studies are required to clarify the mechanisms. Although we demonstrated that shear stress enhanced osteogenic differentiation of iPSCs (Fig. 2), it upregulated pluripotent markers (Fig. 1E and F, Supplementary Fig. 1). This may happen because iPSCs displayed heterogenicity of the cells in different stages ranging from stem cells to osteogenic cells, in which shear stress plays different roles on them^7,8^. This necessitates further investigation on the role of shear stress on each specific osteogenic-lineage cell type along with osteogenic differentiation of iPSCs.

Fluid shear stress influences osteoblast cell behaviors, that is, differentiation and maturation, depending on the force magnitude and application duration. Shear stress has been reported to promote the osteogenic differentiation and maturation of osteoblastic cells derived from MSCs^32,33^. High-force magnitudes appear to induce nitric oxide (NO), adenosine triphosphate (ATP), prostaglandin E2 (PGE2), and Opn production by pre-osteoblastic cells^34^ and osteocytes^35^. Previous reports showed that the duration of shear force application affects the osteogenic differentiation of pre-osteoblastic cells and MSCs^36^. Therefore, we further investigated the effects of shear stress on the osteogenic differentiation of iPSCs over a shorter period, that is, 12 h and 24 h, respectively. The control static culture in OS medium was collected at the same time point for evaluation. Enhanced expression of osteogenic genes *Osx, Col1a1, Ocn, Opn* was observed under 24 h of shear stress application compared to static culture (Fig. 3A). When compared to static culture, these genes were further upregulated by shear loading for 48 h (Fig. 3A), corresponding to the increase in mineral deposition on the outgrowth cells (Fig. 3B and C). These suggested that 24 h of shear stress application is the optimal time point for enhanced osteogenic differentiation but not mineralization. Taken all evidence, shear stress could effectively induce the osteogenic cell commitment of iPSCs within 24 h; this could be confirmed by the enhanced mineralization in the shear stress application group for 1 day and maintained in static culture for a further 5 days (Fig. 5D and E).

The mineral deposit was observed on day 3 of osteogenic induction of iPSCs in static culture, and shear stress loading for 2 days further enhanced the mineralization (Fig. 3B and C). This was different from osteogenic differentiation of MSCs which is a week-long event, and no mineralization can be observed within 3 days of osteogenic differentiation. As our previous report^37^ showed mineral deposition of the iPSC-mediated osteogenesis on day 3. In addition, we also confirmed that the mineral deposit occurred after osteogenic differentiation as there was no mineral deposit on day 0 and day 1 of osteogenic induction (data not shown). This may suggest that iPSC-mediated osteogenesis might differ from general MSC osteogenesis, particularly in the mineralization process as cell aggregates. Master transcriptional regulators of early osteogenic differentiation are Runx2 and Osx. The previous report showed Runx2 expression in nonskeletal tissue such as sperm and brain^38^. In contrast, *Osx* knockout mice demonstrated complete abrogation of bone formation^38^. This suggests that Osx is a specific transcriptional factor for osteogenic differentiation. Indeed, the enhanced osteogenic differentiation of iPSCs induced by shear loading was, in part, confirmed based on the expression and nuclear localization of Osx (Fig. 3D and E) as well as Opn protein levels (Fig. 3F and G). In addition, our demonstration that pretreatment osteogenically-induced iPSCs with shear stress for 2 days could enhance in vivo bone formation (Supplementary Fig. 2) would support the enhanced in vitro osteogenesis of iPSCs by shear stress. This may suggest utilizing shear stress as an effective factor for the improvement of in vitro tissue-engineered bones for regenerative bone therapy.

Various mechanisms have been reported as involved in the promotion of stem cell osteogenic differentiation by mechanical stimuli, depending on the cell type and loading force. For instance, intermittent compressive force promoted the osteogenic differentiation of human PDLSCs via the transforming growth factor-beta (TGF-β) pathway^5^. Mechanical stretch promoted the osteogenic differentiation of human jaw bone marrow-derived MSCs via NF-kB pathway suppression^39^. Mechanical tension promoted the osteogenic differentiation of human PDLCs via Cx43 and downstream Erk1/2 activation^40^. Indeed, we detected enhanced expression of Cx43 in the shear stress loading group compared to that in the static group (Fig. 4A and B). In addition, the highest Cx43 expression was observed in the 0.5 Pa group. Moreover, we demonstrated that Erk1/2 activation was enhanced by shear stress, and 0.5 Pa induced the greatest Erk1/2 activation in parallel to the highest Cx43 expression. As signaling downstream of Cx43 occurs through Erk1/2, our findings suggest their involvement in enhancing the osteogenic differentiation of iPSCs.

**Figure 6:**
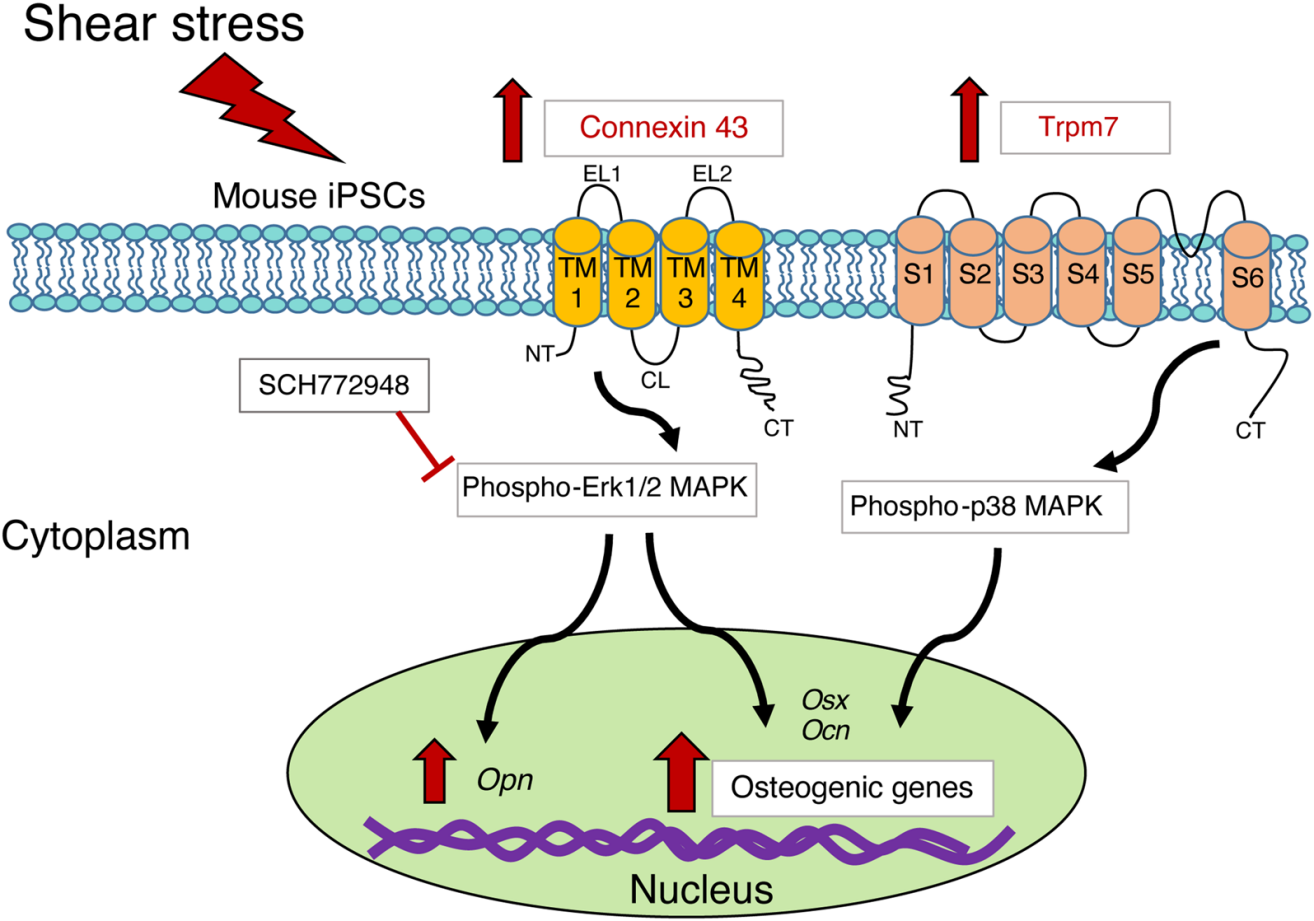
Schematic diagram of shear stress-enhanced osteogenic differentiation of mouse iPSCs and the relevant underlying mechanisms. Shear stress enhanced osteogenic differentiation of mouse iPSCs through Connexin 43 (Cx43) and Erk1/2 signaling. The inhibition of Erk1/2 activity partly suppressed osteogenic gene expression. Trpm7 expression and downstream phosphorylated-p38 were upregulated under shear stress implying that they might be involved in the enhanced osteogenic differentiation of iPSCs by shear stress.

We further confirmed the involvement of Erk1/2 activity in shear stress-enhanced osteogenic differentiation of iPSCs. The cells were treated with 10 μM SCH772984^41^ to inhibit Erk1/2 activity during shear force application. The downregulation of *Osx,* a master transcriptional factor as well as bone extracellular matrix-related genes *Ocn* and *Opn* was observed under inhibitor treatment (Fig. 5C). In addition, the inhibition of Erk1/2 during shear stress application for one day inhibited the enhanced mineralization (Fig. 5D). These results suggested that the shear stress-enhanced osteogenic differentiation of iPSCs possibly occurred through Erk1/2 signaling. Under shear loading, the partial suppression of osteogenic genes via Erk1/2 inhibitor treatment may suggest the involvement of other mechanisms (Fig. 6). As a previous report demonstrated that shear stress enhanced the osteogenic differentiation of mouse MSCs via activation of the Trpm7 channel and downstream p38 signaling^16^, we confirmed the upregulation of Trpm7 as well as p38 activation (Fig. 4), highlighting their association with the shear stress-enhanced osteogenic differentiation of iPSCs. However, it is possible that partial inhibition of osteogenic gene expression might be due to the incomplete abrogation of Erk1/2 phosphorylation by SCH772984 (Supplementary Fig. 3A). As the phosphorylation of Erk1/2 is carried out by various kinases, and SCH772984 inhibits Erk1/2 activity by targeting RAF and MEK kinases, the use of other Erk1/2 inhibitors to confirm its involvement is necessary. Thus, this should be addressed in future studies.

Erk1/2 a member of the mitogen-activated kinase (MAPK) family, with MAPK signaling acting as a master regulator of cell behavior and fate^42^. It phosphorylates both cytosolic and nuclear substrates to activate or inhibit their activity^43^. Further, active Erk1/2 phosphorylates Runx2 at serine residues, including Ser301 and Ser319, to regulate osteogenic differentiation^44^. This leads to an increase in Runx2 binding to cofactors such as CREB as well as CBP/p300 and promotes the expression of target genes. An increase in cytoplasmic phosphorylated Erk1/2 has been associated with upregulated nuclear Runx2 expression, which implies that Erk1/2-induced Runx2 phosphorylation occurs in the cytoplasm^45^. In addition, active cytoplasmic Erk1/2 can phosphorylate RSK2, which in turn phosphorylates ATK4 to regulate several aspects of osteoblast biology, ranging from the onset of differentiation to osteoblast-specific gene expression^46^. Apart from Runx2, Erk1/2 has been reported to regulate Osx at both the transcriptional and translational levels. Overexpression of constitutively active MEK to activate Erk1/2 upregulated the mRNA and protein levels of Osx, increased Osx protein stability, and enhances its transcriptional activity during osteoblast differentiation^47^. However, the detailed mechanisms remain unclear. These findings underscore the importance of cytoplasmic active Erk1/2 in phosphorylating master transcription factors for the osteogenic differentiation of cells. Indeed, our observation that shear stress enhanced Erk1/2 phosphorylation, which accumulated in the cytoplasmic region (Fig. 4C), and induced the osteogenic differentiation of iPSCs, could be partly supported by the above-presented notion.

As severe bone loss exceeds the natural bone-healing capacity, stem cell-based regenerative bone therapy is required. Recent advances in tissue engineering have pushed stem cell-based regenerative medicine forward. An optimal mechanical loading microenvironment, similar to the in vivo bone microenvironment, is critical for bone tissue engineering. Fluid shear stress is widely used in studies of bone tissue engineering of MSCs to promote their osteogenic differentiation^48^. However, iPSC differentiation has remained unclear. Indeed, our study first demonstrated that 0.5 Pa was an optimal shear force magnitude for enhancing the osteogenic differentiation of mouse iPSCs, with the resulting osteogenic cells and tissues, expecting to enhance in vivo bone formation. As the manner in which shear stress is applied, that is, continuous or intermittent, influences the osteogenic differentiation ability of human MSCs^48^, to our culture system can be employed for elucidating the effects of different application modes. We expect that Shear stress (0.5 Pa) effectively promoted the osteogenic differentiation of mouse iPSCs representing a key step in the application of shear stress loading technology to improve iPSC-based tissue engineering for regenerative bone therapy.

In conclusion, the present study found that shear stress enhances the osteogenic differentiation of mouse iPSCs, in part through Cx43 and downstream Erk1/2 signaling. In addition, 0.5 Pa would be the optimal shear force magnitude for the induction of mouse iPSC differentiation toward osteogenic cells.

## Methods

### iPSC culture

In a 6-well culture plate, mouse gingival fibroblast-derived iPSCs (7 × 10^3^ cells/well) were seeded on inactivated SNLP76.7-4 feeder cells and maintained in ES medium consisting of Dulbecco’s modified Eagle’s medium (DMEM with 4.5 g/L glucose and without sodium pyruvate; Nacalai Tesque), 15% FBS (Thermo Fisher Scientific, Grand Island, NY, USA), 2 mM l-glutamine (Wako Pure Chemical), 1 × 10^–4^ M nonessential amino acids (Thermo Fisher Scientific), 1 × 10^–4^ M 2-mercaptoethanol (Thermo Fisher Scientific), and penicillin (50 U)/streptomycin (50 µg/mL) (Wako Pure Chemical)^15^.

### Osteogenic induction of iPSCs

iPSCs were trypsinized by adding 500 µL 0.25% trypsin and 1 mM EDTA (Wako Pure Chemical) to each well in order to remove SNLP76.7-4 feeder cells. Then, 500 µL of trypsin solution was added to iPSCs with gentle pipetting to collect iPSC clusters. The suspension of iPSC clusters was then transferred to a low-attachment 10 cm dish using the ratio of a well of 6-well plate (Greiner Bio-One, Frickenhausen, Germany) to a 10-cm dish (Thermo Scientific), maintained for two days to form EBs. The medium was replaced with ES medium supplemented with 1 µM all-trans retinoic acid (RA; Wako Pure Chemical) and maintained for another two days prior to adherent culture in 35-mm dishes (Corning)^14^ for another day. The culture medium was then replaced by osteogenic induction medium, consisting of α-MEM (Nacalai Tesque) supplemented with 15% fetal bovine serum (FBS: Thermo Fisher Scientific), 0.01 μM dexamethasone (Sigma-Aldrich, St. Louis, MO, USA), 10 mM β-glycerophosphate (Sigma-Aldrich), 50 μg/mL ascorbate-2-phosphate (Sigma-Aldrich), and 1% antibiotic–antimycotic solution (100 U/mL penicillin, 100 μg/mL streptomycin, and 250 ng/mL amphotericin B; Thermo Fisher Scientific)^3^.

### Shear force application

Cells in osteogenic induction medium (2 mL/35-mm dish) were maintained for one day. The osteogenic induction medium was then changed to 4 mL/35-mm dish prior to shear stress application^14^ for 2 days (Fig. 1B). A novel shear stress loading apparatus (Thai patent No. 1801000629) was used to apply shear force on the cells via the angular flow of the medium. By controlling rotational speed of the rotating rod of the apparatus, we could vary the desired shear stress, which was estimated by using computational fluid dynamics (CFD) simulation. Three different rotational speeds of 145, 300, 610 rpm were used in this study to generate the area-averaged shear stress of 0.15, 0.5 and 1.5 Pa, respectively. The homogeneity of the applied shear stress on cells is expressed in terms of the uniformity index, which was 0.73–0.82^14^. More information about the geometry of the rotating rod, shear stress distribution, magnitude, and uniformity index at different rotational speeds can be found in our previous work^14^. The control group was not subjected to force or static culture. To study the involvement of Erk1/2 signaling in the stress-induced osteogenic differentiation of iPSCs, cells were treated with 10 μM SCH772984 (Cayman Chemical). The inhibitor was added to the medium 6 h before shear stress application and was maintained in the medium during shear stress application for 24 h.

### Cell viability assay

Cell viability assays were performed using the WST-1 based colorimetric reagent (Sigma Aldrich). To measure cell proliferation after shear stress application for 48 h, the 35-mm culture dish was incubated at 37 °C for 1 h with 1.5 mL medium containing WST-1 solution (1:10 final dilution). Fresh medium was used as a background control (blank). The amount of formazan product was measured using a microplate reader at 420 nm. The reference wavelength used in this study was 630 nm. For the standard curve, Day 4 EBs were trypsinized and then aliquoted into a series of cell concentrations, including 1 × 10^5^, 2 × 10^5^, 3 × 10^5^, 4 × 10^5^, 5 × 10^5^, 7 × 10^5^, and 1 × 10^6^ cells/tube. The cells were maintained in 1.5 mL culture medium containing WST-1 solution for 1 h prior to measurement. To measure the cytotoxic effect of SCH772984, dissociated iPSCs from Day 4 EBs were seeded into a microplate (tissue-culture grade, 96 wells, flat bottom) at a final volume of 100 μL/well culture medium supplemented with 1, 10, 20, 30, 40, and 50 μM SCH772984. The cells were incubated at 37 °C for 24 h prior to incubation in culture medium supplemented with WST-1 reagent for 1 h. The measurement of formazan products was performed at 420 nm with a reference wave length of 630 nm. Fresh medium was used as a blank.

### RT-PCR analysis

Total cellular RNA was extracted using TRIzol reagent (Ambion/Life Technologies, Carlsbad, USA). RNA isolation and purification were performed using a spin column (RNeasy Mini Kit; Qiagen, GmBH, Germany) prior to DNase treatment and removal (DNA-free™ Kit; Thermo Fisher Scientific). Complementary DNA was synthesized from 1 µg of total RNA as previously described^10^. Real-time RT-PCR using the Thunderbird SYBR qPCR Mix (Toyobo, Osaka, Japan) was performed on a StepOnePlus real-time PCR system (Thermo Fisher Scientific). Target gene expression was quantitatively analyzed via the △△CT method and normalized to *Gapdh*. Primer sequences used are listed in Supplementary Table 1. For semi-quantitative RT-PCR, genes of interest were amplified on a thermal cycler (*GeneAtlas G02; Astec* Co., Ltd., Fukuoka, *Japan)* using the GoTaq® Green Master Mix (Promega Corporation, Madison, WI, USA) according to the manufacturer’s instructions^10^. PCR amplification consisted of an initial denaturation for 5 min at 95 °C, followed by 30 s denaturation at 95 °C, annealing for 30 s, and extension for 30 s at 72 °C. The final extension step was performed at 72 °C for 10 min. The PCR products were analyzed via 2% agarose gel electrophoresis with ethidium bromide. Subsequently, the bands of target genes were detected under ultraviolet (UV) light illumination. *Gapdh* was used as an internal control. The primer pairs used for RT-PCR are listed in Supplementary Table 2.

### Immunofluorescent staining

The samples were fixed in a 10% formalin neutral buffer solution (Wako Pure Chemical) for 15 min. After washing, non-specific binding was blocked for 1 h using a blocking buffer containing 2% bovine serum albumin (BSA; Wako Pure Chemical), 0.1% Tween20 (Sigma-Aldrich), and 0.01% Triton-X (Wako Pure Chemical). Next, the samples were incubated with a primary antibody at 4 °C overnight and washed with phosphate buffer saline (PBS). The primary antibodies used in this study included an anti-Opn monoclonal antibody (sc-21742: 1/100, Santa Cruz Biotechnology, CA, USA), anti-osterix monoclonal antibody (F-3, sc-393325: 1/100, Santa Cruz Biotechnology), anti-LTRPC7 monoclonal antibody (H-4, sc-271099:1/100, Santa Cruz Biotechnology), anti-Cx43 (F-7, sc-271837: 1/200, Santa Cruz Biotechnology), anti-phospho-Erk1/2 (9101S: 1/500, Cell Signaling Technology), and control IgG [normal mouse IgG (sc-2025): 1/50 or rabbit IgG (sc-2027): 1/50, Santa Cruz Biotechnology]. Samples were then incubated with an Alexa Fluor 488-conjugated goat anti-mouse IgG (1/500, Molecular Probes, Thermo Fisher Scientific) or an Alexa Fluor 488-conjugated donkey anti-rabbit IgG (1/500, Abcam) for 1 h at room temperature. Actin-stain 555 phalloidin (1:500, Cytoskeleton) was used to stain F-actin. Nuclear staining was performed using Hoechst 33,258 (1/500, Thermo Fisher Scientific, MA, USA). The samples were then washed with PBS, and staining was assessed under a fluorescent microscope (LSM780, Zeiss)^38^. The 3–4 fields were randomly picked for the quantification of relative mean fluorescence intensity by ImageJ software.

### Western blot analysis

After washing with PBS, the cells were lysed via ultrasonic homogenization in RIPA buffer (Wako Pure Chemical) supplemented with a protease inhibitor (cytoskeleton) and phosphatase inhibitors (Nacalai Tesque). The protein concentration of each sample was measured using a protein colorimetric assay (Pierce 660 nm Protein Assay; Thermo Fisher Scientific). Protein samples (30 μg) were loaded onto a 12% polyacrylamide gel (SDS-PAGE) for electrophoresis and then transferred onto a polyvinylidene fluoride membrane (Bio-Rad Laboratories, CA, USA). After blocking with 5% skim milk (Wako Pure Chemical) in TBST, the samples were incubated with an anti-Opn monoclonal antibody (sc-21742: 1/200, Santa Cruz Biotechnology), anti-Osx monoclonal antibody (F-3, sc-393325: 1/200, Santa Cruz Biotechnology), and anti-LTRPC7 monoclonal antibody (H-4, sc-271099: 1/200, Santa Cruz Biotechnology), anti-Col1a1 (NBP1-30054: 1/1000, NOVUSBIO), anti-Cx43 (F-7, sc-271837: 1/200, Santa Cruz Biotechnology), anti-phospho-Erk1/2 (9101S: 1/1000, Cell Signaling Technology), anti-phospho-p38 (9211S: 1/1000, Cell Signaling Technology), anti-Oct4 (2840P: 1/1000, Cell Signaling Technology), anti-Klf4 (4038S: 1/1000, Cell Signaling Technology), anti-Nanog (8822S: 1/1000, Cell Signaling Technology), or anti-Gapdh (MAB374: 1/3000, Millipore) at 4 °C overnight. After washing with TBST (10 mM Tris–HCl, pH 7.4, 100 mM NaCl, and 0.1% Tween), the membranes were incubated with a horseradish peroxidase (HRP)-conjugated anti-mouse IgG secondary antibody (sc-516102:1/3000, Santa Cruz Biotechnology) or an HRP-conjugated anti-rabbit IgG secondary antibody (sc-2379:1/3000, Santa Cruz Biotechnology) at room temperature for 1 h. Signals were detected with HRP substrate (Merck Millipore, Burlington, MA) using an image analyzer (ImageQuant LAS-500; GE Healthcare Japan, Tokyo, Japan).

Quantitative analysis of relative protein expression was performed using the ImageJ software (National Institutes of Health, Bethesda, MD, USA). The band density of proteins of interest (n = 3) was normalized to Gapdh expression.

### ARS staining

Calcium deposition was evaluated via ARS staining^49^. The cells were washed with PBS before fixation with 10% formalin in phosphate buffer. The cells were then incubated in 40 mM Alizarin Red S (Sigma) solution for 20 min with gentle shaking, washed with distilled water, and left to dry before obtaining digital images.

For quantitative analysis, samples were incubated in 10% acetic acid at room temperature for 30 min. The samples were then scraped and transferred into a 1.5-mL Eppendorf tube. Mineral oil was added to each sample before heating for 10 min at 85 °C. After cooling on ice for 5 min, samples were centrifuged at 20,000 × g for 15 min. The colored supernatant was collected in a new tube, and 10% ammonia was added. Subsequently, the optical density of the supernatant was measured at a wavelength of 405 nm.

### Animal experiment

The reporting in the manuscript follows the recommendations in the ARRIVE guidelines. All animal experiments in this study strictly followed a protocol approved by the Animal Research Subjects Committee of Tohoku University (approval number: 2018DnA-002). All methods were carried out in accordance with relevant guidelines and regulations. This study used a total of 7-male 10-week-old Sprague–Dawley (SD) rats (Nippon SLC, Shizuoka, Japan). The animals were subjected to anesthesia prior to the creation of right and left defects (5 mm in diameter) on parietal region of rat calvaria^38^. The anesthetics for injection in the rat were 1 mg/mL medetomidine hydrochloride (Domitol, Meiji Seika Pharma Co., Ltd., Tokyo, Japan), 5 mg/mL midazolam (Dormicum, Astellas Pharma Inc., Tokyo, Japan), and 5 mg/mL butorphanol (Vetorphale, Meiji Seika Pharma Co., Ltd.) in 0.9% w/v Sodium chloride (normal saline) (Otsuka, Pharma Co., Ltd., Tokyo, Japan)^50^. The rats were maintained under isoflurane gas during operation. The right and left defects were filled up with living iPSCs prepared from static and shear-loading cultures, respectively. After the operation had finished, the rats were injected with 5 mg/mL atipamezole (Antisedan, Orion Corporation, Finland) in 0.9% w/v Sodium chloride to reverse the anesthesia effect. The rats were subcutaneously injected daily with Cyclosporine A (LC Laboratories, Woburn, MA, USA) to prevent immune rejection of xenogeneic mouse cells implanted. After transplantation for 3 weeks, the rats were sacrificed and calvarias were extracted entirely. After fixing calvarias with 10% formalin neutral buffer solution (Wako Pure Chemical) overnight, micro computed tomography (CT) analysis was performed.

### Micro CT and bone morphometric analyses

Bone mineral density (BMD) of newly formed bone was determined using ScanXmate-E090 three-dimensional micro X-ray CT imaging device (Comscan Tecno Co., Ltd., Kanagawa, Japan) and TRI/3D-BON bone structure analysis software (Ratoc System Engineering, Tokyo, Japan). An energy level of 83 kVp and a current of 63 μA were used to X-rayed calvarias through a 1-mm-thick brass filter with the 42 µm/pixel isotropic voxel size. 3D images were reconstructed using the calibration curve of bone mineral content obtained by the scanning of hydroxyapatite phantom under the same X-ray conditions. The density of new bone formation at the selected 5-mm round-shape defect site was analyzed using the specific thresholds for bone tissue, which were determined by superimposing segmented images over the original grayscale X-ray images.

### Statistical analyses

One-way analysis of variance (ANOVA) with the Tukey or Dunnett post hoc test was used to compare more than two groups. A value of *P* < 0.05 was defined as indicative of statistical significance. The Student’s *t*-test was performed to compare the two groups. *P* < 0.05 was considered statistically significant.

## Supplementary Information

Supplementary Information.

## Data Availability

The datasets generated and/or analyzed during the current study are available from the corresponding author upon reasonable request.
