# Supplementary material for: Application of shear stress for enhanced osteogenic differentiation of mouse induced pluripotent stem cells
Source: Sci Rep. 2022 Nov 8;12:19021. doi: 10.1038/s41598-022-21479-8 (PMC9643422; doi:10.1038/s41598-022-21479-8)
Supplement: Supplementary file 1 — Supplementary Information. [file 41598_2022_21479_MOESM1_ESM.pdf]

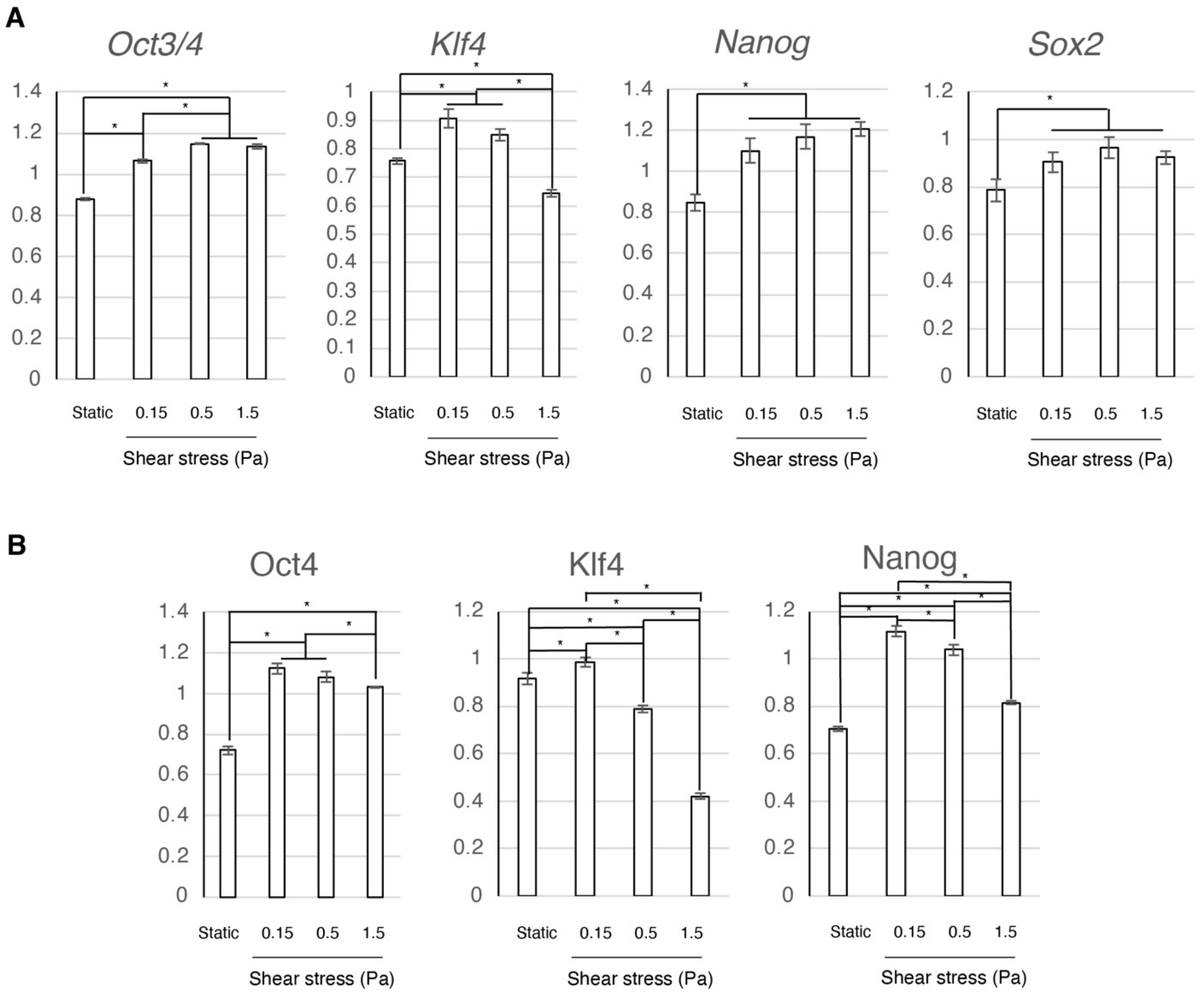

Supplementary Figure 1. Continuous shear stress influenced pluripotency of iPSCs. Quantitative analysis of relative gene (**A**) and protein (**B**) expression of pluripotent markers by ImageJ software. The expression of pluripotency genes and proteins was normalized by Gapdh gene and protein, respectively. *: $P<0.05$, ANOVA with Tukey's multiple comparison test. Data represent the mean ± SD (n=3).

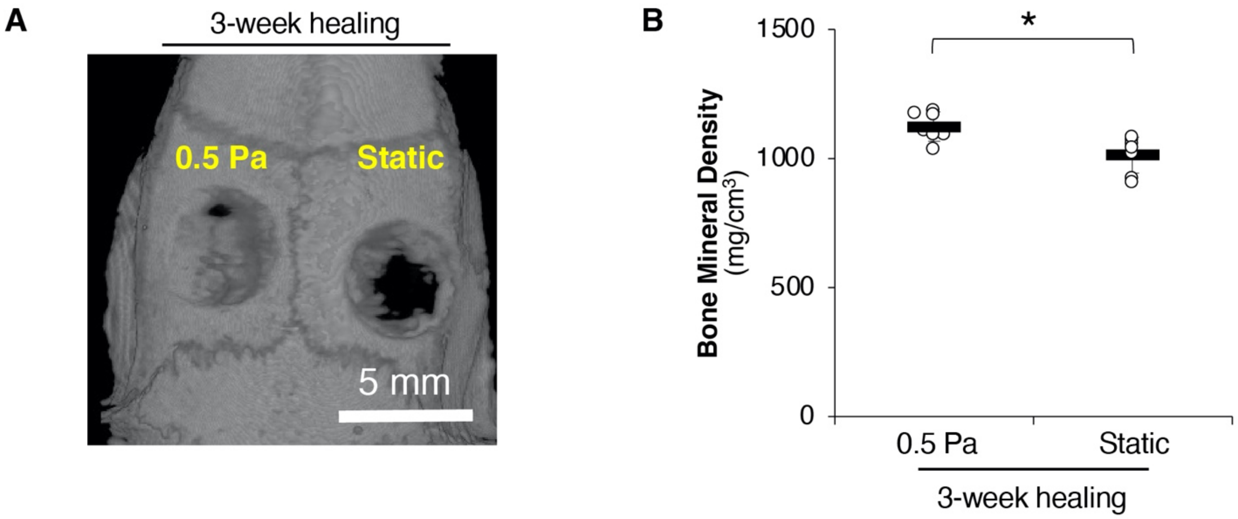

Supplementary Figure 2. Pretreatment osteogenically-induced iPSCs with shear stress induced *in vivo* bone formation in critical size defects. After healing for 3 weeks, new bone formation was evaluated using micro CT and bone morphometric analyses. (**A**) Representative 3D reconstruction image from micro CT showing new bone formation in the defect area. (**B**) New bone formation was quantitatively analyzed by bone morphometry. *: $P<0.05$, Student's *t*-test. The data represent the mean ± SD (n=7).

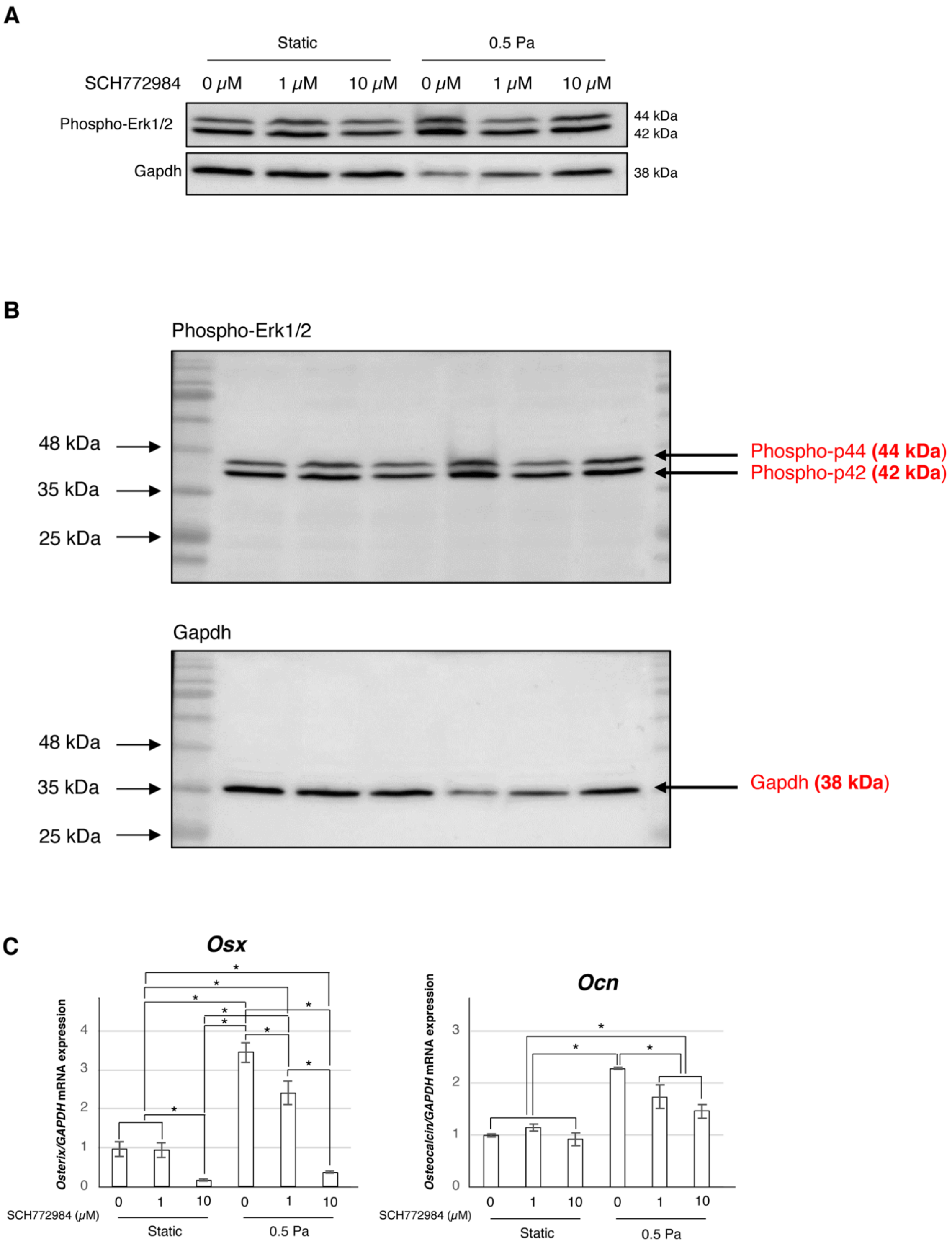

Supplementary Figure 3. Effects of different concentrations of Erk1/2 inhibitor on the osteogenic differentiation of iPSCs under static and shear stress culture. (**A**) Western blotting analysis of phospho-Erk1/2 levels under treatment with SCH772984, an Erk1/2 inhibitor. (B) Raw data of phospho-Erk1/2 and Gapdh band detection (**C**) Real-time RT-PCR analysis of the expression of osteogenic genes *Osx*, a master transcriptional factor for osteogenesis, and *Ocn*, an abundant protein required for mineralization. *: $P<0.05$, ANOVA with Tukey's multiple comparison test. Data represent the mean ± SD (n=3).

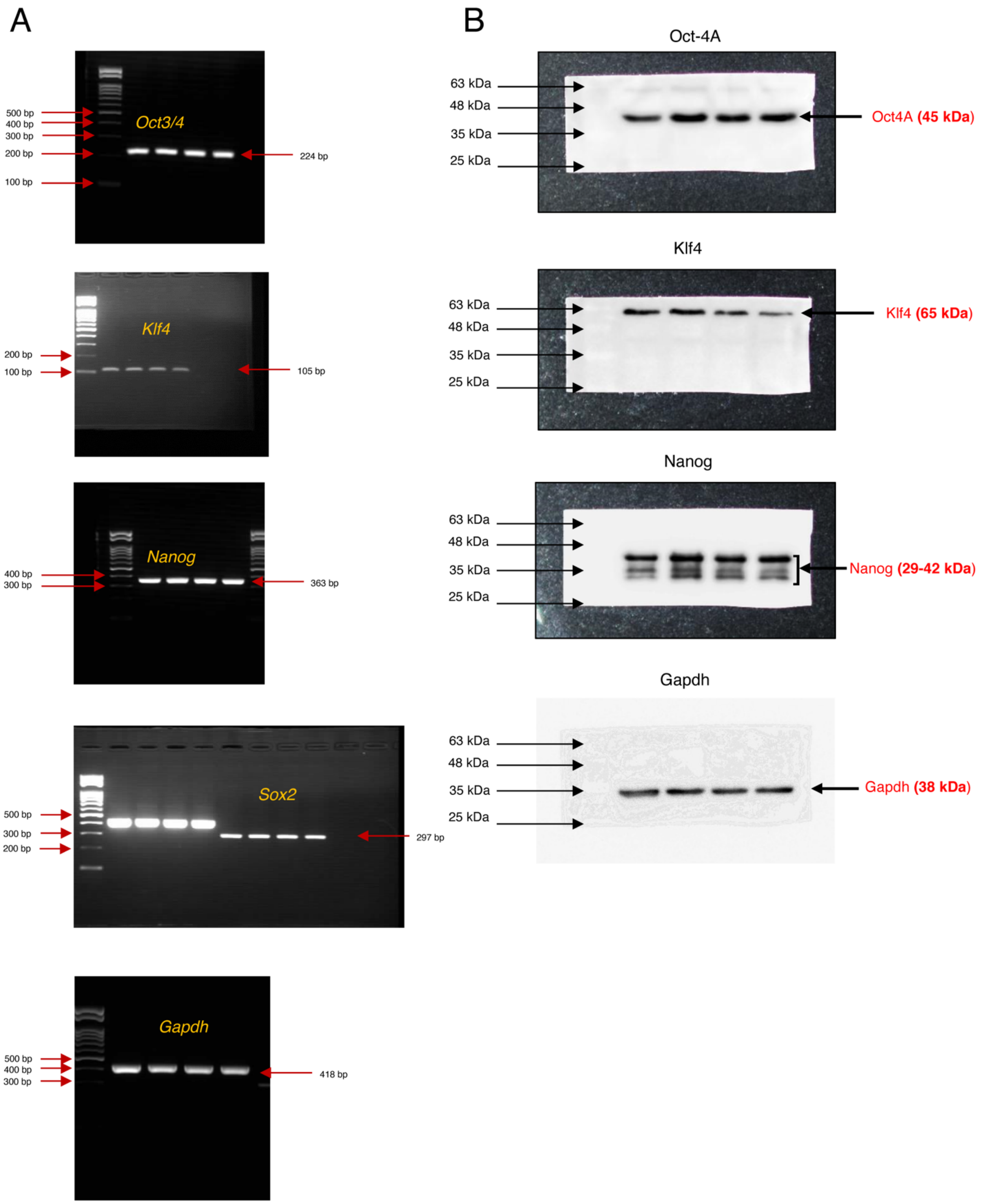

Supplementary Figure 4. Raw data of pluripotency marker genes and proteins using (**A**) PCR in Fig. 1E and (**B**) western blotting analysis in Fig. 1F, respectively.

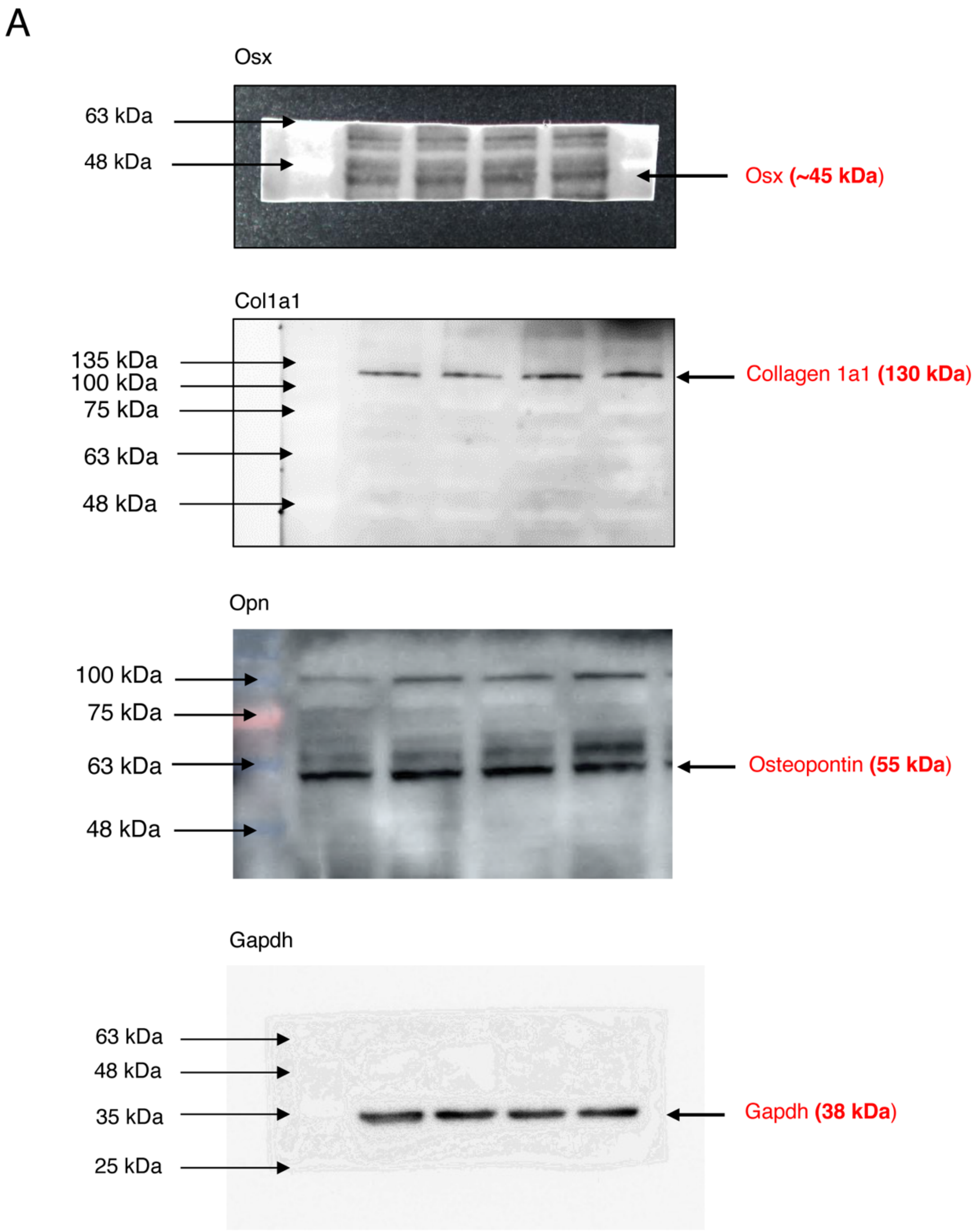

Supplementary Figure 5. (A) Raw data of the expression of osteogenic proteins, as demonstrated in Fig. 2B

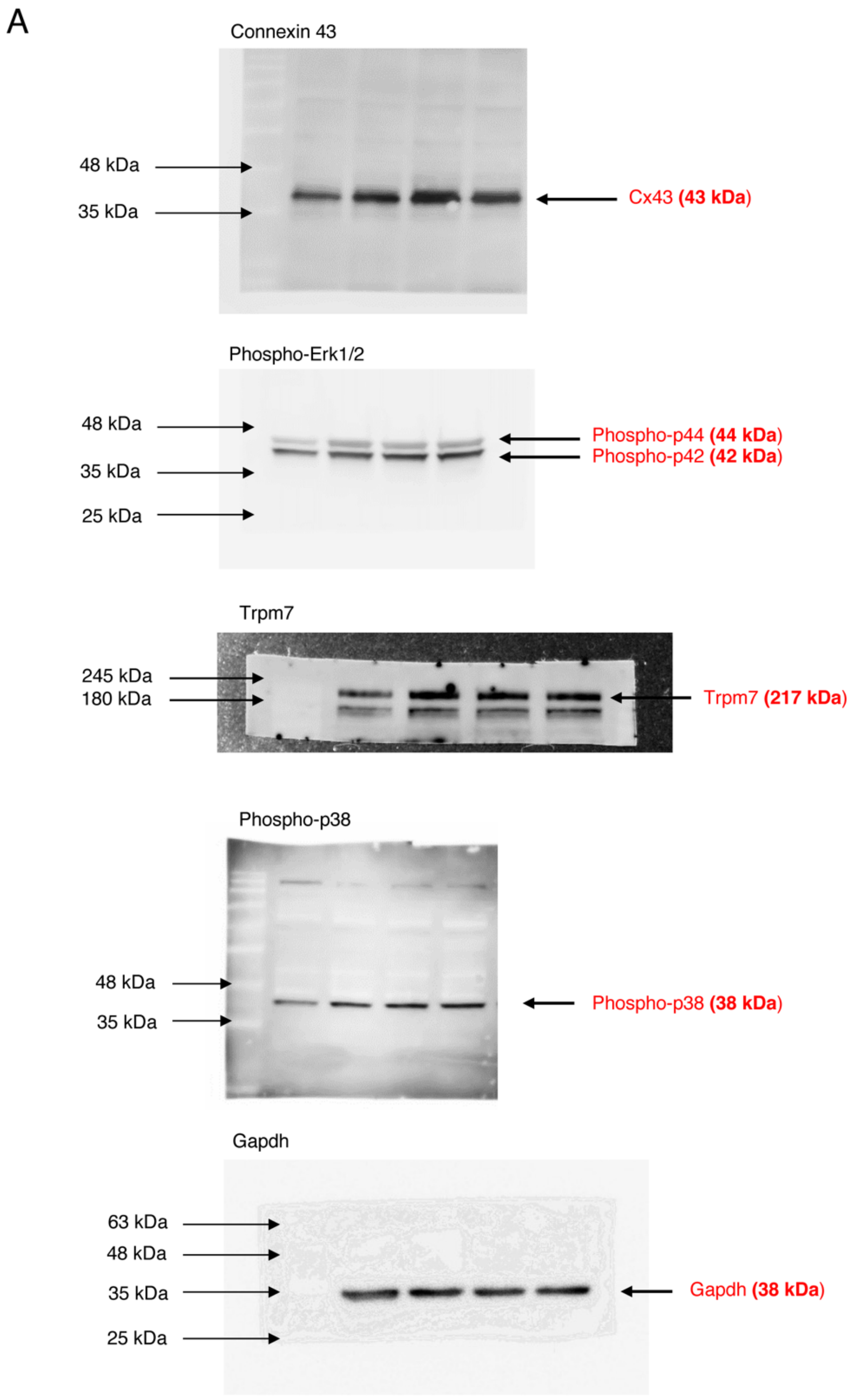

Supplementary Figure 6. (**A**) Raw data of western blot analysis of cell surface channels Connexin 43 (Cx43) and Trpm7 as well as the phosphorylation of downstream factors including Erk1/2 (phospho-Erk1/2) and p38 (phospho-p38) as demonstrated in Fig. 4A.

Supplementary Table 1: Primers used for SYBR Green real-time RT-PCR

Gene name	Primers (Fw, forward; Rv, reverse)	Product size (bp)	Accession number
Runx2	Fw: 5'-CGGGCTACCTGCCATCAC-3'<br>Rv: 5'-GGCCAGAGGCAGAAGTCAGA-3'	78	NM_001146038.2
Osterix (*Sp7*)	Fw: 5'-CTCGTCTGACTGCCTGCCTAG-3'<br>Rv: 5'-GCGTGGATGCCTGCCTTGTA-3'	84	NM_130458.3
Collagen 1a1	Fw: 5'-TGTCCCAACCCCCAAAGAC-3'<br>Rv: 5'-CCCTCGACTCCTACATCTTCTGA-3'	92	NM_007742.3
Osteocalcin (*Bglap*)	Fw: 5'-CCGGGAGCAGTGTGAGCTTA-3'<br>Rv: 5'-AGGCGGTCTTCAAGCCATACT-3'	68	NM_007541.3
Osteopontin (*Spp1*)	Fw: 5'-TCTCCTTGCGCCACAGAATG-3'<br>Rv: 5'-TCCTTAGACTCACCGCTCTT-3'	399	NM_001204201.1
Gapdh	Fw: 5'-TGCACCACCAACTGCTTAG-3'<br>Rv: 5'-GGATGCAGGGATGATGTTC-3'	177	NM_001289726.1

Supplementary Table 2: Primers used for conventional RT-PCR

Gene name	Primers (Fw, forward; Rv, reverse)	Product size (bp)	Accession number
Oct3/4	Fw: 5’-TCTTTCCACCAGGCCCCCGGCTC-3’<br>Rv: 5’-TGCGGGCGGACATGGGGAGATCC-3’	224	NM_013633.3
Sox2	Fw: 5'-TAGAGCTAGACTCCGGGCGATGA-3'<br>Rv: 5'-TTGCCTTAAACAAGACCACGAAA-3'	297	NM_011443.4
Klf4	Fw: 5'-TCTCAAGGCACACCTGCGAA -3'<br>Rv: 5'-TAGTGCCTGGTCAGTTCATC -3'	105	NM_010637.3
Nanog	Fw: 5'-AGGGTCTGCTACTGAGATGCTCTG-3'<br>Rv: 5'- CAACCACTGGTTTTTCTGCCACCG-3'	363	NM_028016.3
Gapdh	Fw: 5’-CACCATGGAGAAGGCCGGGG-3’<br>Rv: 5’-GACGGACACATTGGGGGTAG-3’	418	M32599
